# Rethinking biological biomarkers to track treatment efficacy in Alzheimer's disease: Focus on brain connectivity

**DOI:** 10.1002/alz.70083

**Published:** 2025-03-20

**Authors:** Lorenzo Pini, Bruno P Imbimbo

**Affiliations:** ^1^ Department of Neuroscience University of Padova Padova Italy; ^2^ Padova Neuroscience Center University of Padova Padova Italy; ^3^ Department of Research and Development Chiesi Farmaceutici Parma Italy

**Keywords:** Alzheimer's disease, biomarkers, brain functional connectivity

1

In a recent issue of *Alzheimer's & Dementia*, Bittner and colleagues published a comprehensive evaluation of brain positron emission tomography (PET) and cerebrospinal fluid (CSF) biomarkers from two, 116‐week, double‐blind, placebo‐controlled Phase 3 studies (GRADUATE I and II) investigating gantenerumab, an anti‐amyloid beta (Aβ) monoclonal antibody. The studies included 1965 participants with early Alzheimer's disease (AD).^1^ The authors measured a wide range of imaging and fluid biomarkers, including markers of amyloid pathology (amyloid PET, Aβ40, Aβ42), tau pathology (tau PET, p‐tau181, p‐tau217), neurodegeneration (volumetric magnetic resonance imaging (MRI), total tau, neurofilament light chain [NfL]), synaptic dysfunction (neurogranin, neuronal pentraxin‐2 [NPTX2], and alpha‐synuclein [α‐syn]), and glial activation and neuroinflammation (soluble triggering receptor expressed on myeloid cells 2 [sTREM2], glial fibrillary acidic protein [GFAP], S100 calcium‐binding protein B [S100B], and chitinase‐3‐like protein 1 [YKL‐40]). Amyloid PET measurements were performed in 237 participants, tau PET in 201 participants, MRI volumetric assessments in 1952 participants, and CSF biomarker assessments in 315 participants.^1^ Treatment with gantenerumab significantly affected Aβ‐PET burden and CSF levels of Aβ40, Aβ42, total tau, p‐tau181, GFAP, neurogranin, S100B, NfL, α‐syn, and NPTX2. Although the effects of gantenerumab on these biomarkers were consistent with putative effects on brain Aβ deposition, neurodegeneration, and neuroinflammation, the drug did not show significant cognitive, clinical, or functional benefits for patients.^2^ The apparent discrepancy between biomarker improvements and lack of clinical efficacy raises an important question: why does gantenerumab, despite positively influencing several AD biomarkers, fail to translate these effects into cognitive or functional benefits?

This discrepancy is not unique to gantenerumab. Other anti‐Aβ monoclonal antibodies have shown similar inconsistencies. Lecanemab, approved by the U.S. Food and Drug Administration (FDA) in 2023 for early AD, significantly slowed cognitive and clinical decline but did not significantly affect CSF NfL levels, a marker of neural injury.^3^ Similarly, donanemab, another FDA‐approved anti‐Aβ monoclonal antibody, reduced the rate of cognitive and clinical decline^4^ but did not significantly decrease plasma NfL levels compared to placebo,^5^ whereas its effects on CSF NfL levels were not investigated. In addition, its impact on CSF Aβ40 or Aβ42 remains unknown.

Given these findings, we must question whether traditional AD biomarkers, tracking Aβ load, neurodegeneration, and neuroinflammation, are reliable predictors of cognitive and functional outcomes. Recently, we proposed that brain connectivity could represent a promising and underutilized metric for evaluating the functional impact of disease‐modifying therapies.^6^ Brain connectivity recapitulates the hierarchical organization of large‐scale neural networks that govern cognitive function. Efficient communication within and between these networks is highly predictive of behavior in both health and disease,^7^ making it a valuable transdiagnostic tool. For instance, the default mode network, a network[Fig alz70083-fig-0001] encompassing a set of regions from the parietal, frontal, and temporal cortices, shows early vulnerability in AD patients compared to the control counterpart (Figure 1) and exhibits signs of alterations even in the absence of symptoms.^8^

**FIGURE 1 alz70083-fig-0001:**
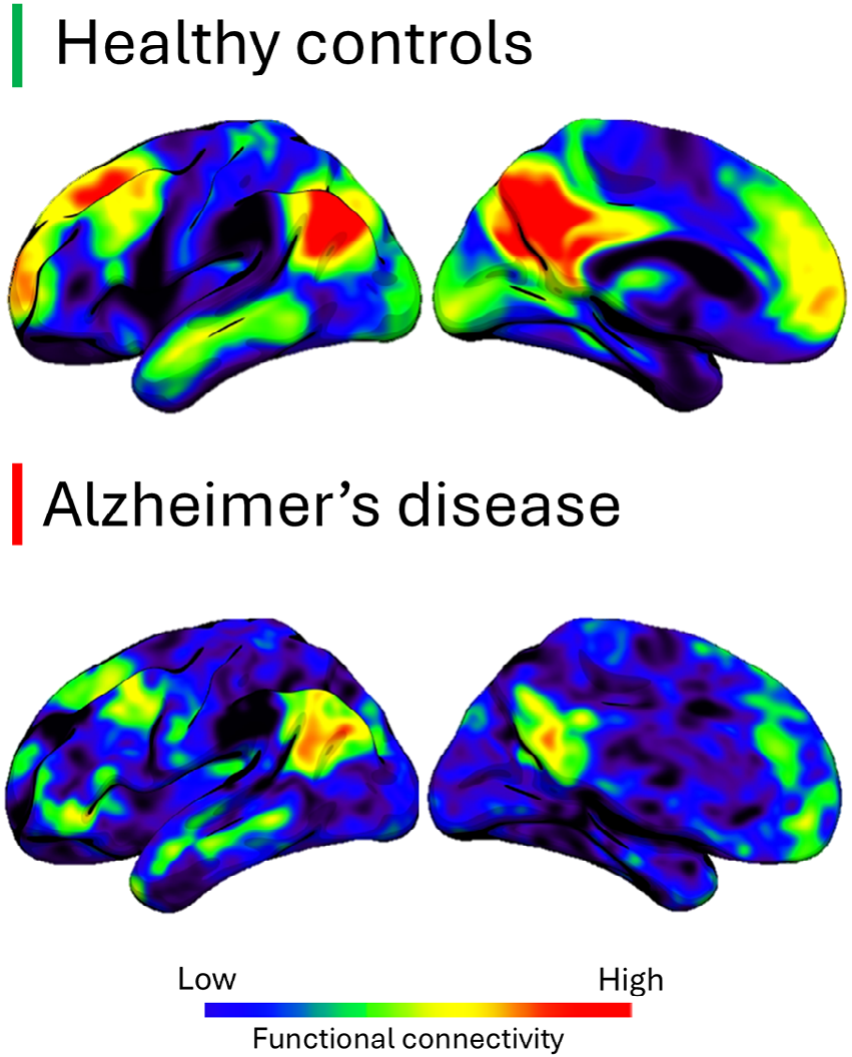
The default mode network in Alzheimer's disease. Exemplary maps from a healthy control (top panel) and a patient with mild Alzheimer's disease (bottom panel) depicting the default mode network. The patient exhibits reduced connectivity (synchronization) between different brain regions compared to the control, highlighting the early vulnerability of this network to pathophysiological changes.

Analyzing brain connectivity changes following treatment could complement biomarker assessments like those performed in Bittner's study. Although the clinical role of connectivity outcomes has yet to be fully recognized, these metrics represent a new paradigm for assessing pharmacological effects in AD trials. This is particularly relevant given the conflicting effects observed with emerging anti‐amyloid and anti‐tau therapies. A marked reduction in pathological markers, such as misfolded protein accumulation or neurodegeneration, does not always correspond to an improvement in overall functioning or disease severity. One possible explanation is that once a critical threshold of pathological burden is surpassed, irreversible alterations in brain plasticity prevent recovery. Connectivity measures may offer insight into this phenomenon by capturing the brain's ability to reorganize and compensate for damage. A similar approach has been applied successfully in brain stroke research, where approximately half of patients experience meaningful recovery while the other half develop long‐term disability. Studies have demonstrated that the restoration (i.e., normalization) of cortical connectivity is a strong predictor of recovery or disability.^9^ Likewise, plasticity mechanisms (reserve, compensation, and maintenance), could help explain individual differences in treatment response, allowing for the identification of patients who are more likely to benefit from specific therapies.

In conclusion, assessing the brain connectivity effects of disease‐modifying treatments could provide a higher level of evidence for cognitive and clinical outcomes. This approach has the potential to reshape pharmacological research by introducing novel surrogate markers, refining patient selection strategies, and guiding the development of new therapies in the AD field.

## CONFLICT OF INTEREST STATEMENT

Lorenzo Pini reported a patent pending (Italian number 102022000015360 and PCT IB2023/057357) for a method using structural disconnections for predicting clinical outcomes. Bruno P. Imbimbo is an employee of Chiesi Farmaceutici. He is listed among the inventors of a number of Chiesi Farmaceutici's patents of anti‐Alzheimer's drugs. Author disclosures are available in the Supporting Information.

## Supporting information



Supporting Information
